# Recent studies on kaempferol and its biological and pharmacological activities

**Published:** 2020-05-13

**Authors:** Jae Kwang Kim, Sang Un Park

**Affiliations:** 1 Division of Life Sciences and Bio-Resource and Environmental Center, College of Life Sciences and Bioengineering, Incheon National University, Incheon 22012, Korea; 2 Department of Crop Science, Chungnam National University, 99 Daehak-ro, Yuseong-gu, Daejeon, 34134, Korea

## ⁯⁯⁯

***Dear Editor,***

Kaempferol (3,5,7-trihydroxy-2-(4-hydroxyphenyl)-4H-chromen-4-one) is a natural flavonol exhibiting different metabolic functions. It is most commonly found in a variety of plants and plant derived foods including grapes, kale, bean, broccoli, tomatoes, spinach, tea, and ginkgo biloba leaves (Cid-Ortega and Monroy-Rivera, 2018[8]; Devi et al., 2015[11]).

The biosynthesis of kaempferol is completed in four major steps. In the first step, a phenylpropanoid metabolic pathway occurs in which phenylalanine is converted into 4-coumaroyl-CoA. Subsequently, 4-coumaroyl-CoA is combined with three molecules of malonyl-coA to form naringenin chalcone (a tetrahydroxychalcone) through the action of chalcone synthase. In the third step, naringenin chalcone is exchanged with naringenin, and its hydroxyl group is involved in the formation of dihydrokaempferol. Finally, dihydrokaempferol, which has a double bond, is converted into kaempferol (Calderón-Montaño et al., 2011[5]; Santos-Buelga et al., 2019[39]).

Several papers have reported the positive effects of dietary kaempferol in reducing the risk of chronic diseases, such as cancer, liver injury, obesity, and diabetes (Imran et al., 2019[20]; Wong et al., 2019[47]). Kaempferol exhibits anti-inflammatory properties and has been used to cure many acute and chronic inflammation-induced diseases, such as intervertebral disc degeneration and colitis, post-menopausal bone loss, and acute lung injury (Ren et al., 2019[37]). Herein, we summarize the most recent published findings on the biological and pharmacological activities of kaempferol (Table 1(Tab. 1); References in Table 1: Adhikary et al., 2018[1]; Alkhalidy et al., 2018[2][3]; Beg et al., 2018[4]; Chen et al., 2020[6]; Chien et al., 2019[7]; Cui et al., 2019[9]; Da et al., 2019[10]; Du et al., 2018[12]; El-Kott et al., 2020[13]; Fernández-Del-Río et al., 2017[14]; Gao et al., 2018[16]; Gao et al., 2019[15]; Gómez-Zorita et al., 2017[17]; Guo et al., 2017[18]; Han et al., 2018[19]; Jiang et al., 2019[21]; Kim et al., 2018[23]; Kim, 2017[22]; Kouhestani et al., 2018[24]; Lei et al., 2019[25]; Li et al., 2017[28]; Li et al., 2018[26]; Li et al., 2019[27]; Liu et al., 2017[29]; Mahobiya et al., 2018[30]; Ming et al., 2017[31]; Moradzadeh et al., 2018[32]; Özay et al., 2019[33]; Pan et al., 2018[34]; Qian et al., 2019 [35]; Rabha et al., 2018[36]; Santos et al., 2019[38]; Sharma and Nam, 2019[40]; Suchal et al., 2017[41]; Torres-Villarreal et al., 2019[42]; Varshney et al., 2017[43]; Vishwakarma et al., 2018[44]; Wang et al., 2018[46]; Wang et al., 2019[45]; Wu et al., 2017[49]; Wu et al., 2018[48]; Yang et al., 2019[50]; Yao et al., 2019[51]; Yeon et al., 2019[52]; Zhang and Ma, 2019[53]; Zhang et al., 2017[55]; Zhang et al., 2019[54]; Zhao et al., 2017[57]; Zhao et al., 2020[56]; Zhong et al., 2018[58]; Zhou et al., 2018[59]; Zhu et al., 2018[60]; Zhuang et al., 2017[61]).

*EXCLI Journal 2020;19:627-634 – ISSN 1611-2156*
*Received: March 15, 2020, accepted: May 05, 2020, published: May 13, 2020*

| Key findings | Reference |
|---|---|
| Kaempferol can provide prophylactic treatment for alcoholic liver injury (AALI) in mice by increasing the level of butyrate receptors, transporters, and tight junction proteins in intestinal mucosa. | Chen et al., 2020 |
| Kaempferol has the ability to protect $CdCl_2$-induced memory deficits and hippocampal apoptosis through its antioxidant potential and the inhibition of Akt/mTOR. | El-Kott et al., 2020 |
| Kaempferol protects retinal ganglion cells from high-glucose-induced injury via extracellular signal-regulated kinases (ERK) and vasohibin-1 signaling. | Zhao et al., 2020 |
| Kaempferol is a promising therapeutic candidate for the treatment of prostate cancer, in which the androgen signaling pathway and vasculogenic mimicry are combined. | Da et al., 2019 |
| Kaempferol shows potential bone-sparing effects by enhancing estrogen signaling followed by activation of the WNT signaling pathway. | Sharma and Nam, 2019 |
| Kaempferol acts as an anticancer agent in MFE-280 endometrial carcinoma cells via apoptosis induction, cell cycle arrest, and inhibition of the m-TOR/PI3K/Akt signaling pathway. | Lei et al., 2019 |
| Kaempferol has a positive role in the development of porcine parthenotes by reducing oxidative stress and increasing mitochondrial function. | Yao et al., 2019 |
| Kaempferol and 5-fluorouracil (5-FU) work synergistically to inhibit cell proliferation and inducing apoptosis in colorectal cancer cells via the suppression of thymidylate synthase or the reduction of p-Akt activation. Therefore, kaempferol and 5-FU may be used together as an effective therapeutic strategy for colorectal cancer. | Li et al., 2019 |
| It is well known that the accumulation of D-ribose induced advanced glycation end-products (AGEs) and reactive oxygen species (ROS) production in mesangial cell causes mitochondrial apoptosis. Kaempferol may reduce these changes and its protective effect could be related to the repair of autophagy. | Zhang et al., 2019 |
| Kaempferol exhibits a protective effect on human aortic endothelial cells (HAECs) against inflammatory injury through the inactivation of nuclear factor κB (NF-κB) and mitogen-activated protein kinase (MAPK) pathways as well as the up-regulation of miR-203. | Cui et al., 2019 |
| Kaempferol has the potential to suppress the proliferation and endorsed autophagy of human gastric cancer SNU-216 cells by up-regulating miR-181a and inactivating the MAPK/ERK (extracellular regulated protein kinases) and phosphatidylinositol 3 kinase (PI3K) pathways. | Zhang and Ma, 2019 |
| Kaempferol shows potential anti-osteoarthritis (OA) effects, exhibiting the down-regulation of miR-146a and repressing the expression of decorin. | Jiang et al., 2019 |
| Kaempferol can be used effectively to reduce lung damage caused by lipopolysaccharide (LPS) by changing TNF receptor associated factor-6 (TRAF6) polyubiquitination. Therefore, kaempferol may act as a novel molecular target to alleviate acute lung injury. | Qian et al., 2019 |
| Kaempferol displays anti-depressive effects, which may be interceded at least in part by its enhanced antioxidant and anti-inflammation effects via the up-regulation of protein kinase B/β-catenin cascade (AKT) activity in the prefrontal cortex of chronic social defeat stress (CSDS) in mice. | Gao et al., 2019 |
| Kaempferol shows neuroprotective effects against striatum injury induced by LPS. The possible mechanisms for its neuroprotective effects involve anti-neuroinflammation, showing blood–brain barrier (BBB) integrity and the down-regulation of the high mobility group box 1(HMGB1)/toll-like receptor 4 (TLR4) pathway. | Yang et al., 2019 |
| 10 μM Kaempferol enhances primordial follicle activation and cell proliferation in the PI3K/AKT pathway and reduces DNA fragmentation in ovine preantral follicles cultured *in vitro*. | Santos et al., 2019 |

EXCLI Journal 2020;19:627-634 – ISSN 1611-2156
Received: March 15, 2020, accepted: May 05, 2020, published: May 13, 2020

| Key findings | Reference |
|---|---|
| Kaempferol prevents hepatocyte apoptosis, protecting mice from liver failure by regulating the endoplasmic reticulum(ER) stress-glucose-regulated/binding immunoglobulin protein 78 (Grp78)-C/EBP-homologous protein (CHOP) signaling pathway. Therefore, kaempferol can be used to treat acute liver failure (ALF). | Wang et al., 2019 |
| Kaempferol amends adipogenic differentiation in 3T3-L1 cells in mature adipocytes by promoting the down-regulation of Cebpa gene expression and decreasing lipid accumulation by exerting its positive effects on the levels of Pnpla2 and Lipe mRNA. From these findings, kaempferol can be considered to exhibit an anti-obesity effect. | Torres-Villarreal et al., 2019 |
| Kaempferol acts as a topical wound healing agent during the treatment of both non-diabetic and diabetic wounds. | Özay et al., 2019 |
| Kaempferol has the ability to inhibit cell migration by targeting extracellular signal-regulated kinases 1/2 (ERK1/2) signaling in human retinal pigment epithelial cells. | Chien et al., 2019 |
| Kaempferol exhibits an anti-inflammatory effect by inhibiting the translocation of cytotoxin-associated gene A (CagA) and vacuolating cytotoxin A (VacA) proteins and enhancing the down-regulation of pro-inflammatory cytokines. | Yeon et al., 2019 |
| Kaempferol shows a protective effect on oxidative stressed-human retinal pigment epithelium (RPE) cell damage due to its antioxidant activity and anti-apoptosis function. Therefore, kaempferol has a potential role in the prevention and therapeutic treatment of age-related macular degeneration (AMD) and other retinal diseases mediated by oxidative stress. | Du et al., 2018 |
| Kaempferol can protect the proliferation, migration, and invasion of hepatic cancer HepG2 cells by down-regulating miR-21 and up-regulating phosphatase and tensin homologue (PTEN) as well as inactivating the PI3K/AKT/mammalian target of rapamycin (mTOR) signaling pathway. | Zhu et al., 2018 |
| Kaempferol reduces oxidized low-density lipoprotein (ox-LDL)-induced apoptosis in human aortic endothelial cells (HAECs) by up-regulating miR-26a-5p via the inactivation of the TLR4/NF-κB signaling pathway, shedding light on the molecular mechanism where kaempferol alleviates ox-LDL-induced endothelial cell (EC) apoptosis. | Zhong et al., 2018 |
| Kaempferol exhibits strong antioxidant activity on erythrocytes as well as inhibitory effects on the growth of cancerous bladder cells by inducing apoptosis and S-phase arrest. | Wu et al., 2018 |
| Kaempferol has the ability to inhibit proliferation, but can also induce apoptosis and autophagy in A549 cells. In addition, kaempferol can up-regulate miR-340, along with PTEN and inactivates the PI3K/AKT pathway. | Han et al., 2018 |
| Kaempferol can be used to promote glucose metabolism in skeletal muscle and inhibits gluconeogenesis in the liver, demonstrating the anti-diabetic action of kaempferol. | Alkhalidy et al., 2018a |
| Kaempferol can activate the inositol-requiring-1 (IRE1)-c-Jun N-terminal kinase (JNK)-CHOP signaling from cytosol to the nucleus and G9a inhibition activates autophagic cell death in gastric cancer (GC) cells. | Kim et al., 2018 |
| Kaempferol can alleviate streptozotocin (STZ)-induced memory impairment in ovariectomized (OVX) rats by enhancing endogenous hippocampal antioxidants, such as superoxide dismutase and glutathione, and reducing neuroinflammation. Therefore, kaempferol can be used as a potential neuroprotective agent against cognitive deficit in Alzheimer's disease (AD). | Kouhestani et al., 2018 |
| Kaempferol has been used to reduce rat pulmonary artery pressure in an endothelium-independent manner via large-conductance $Ca^{2+}$-activated $K^{+}$($BK_{Ca}$) channel, soluble guanylate cyclase (sGC), protein kinase A (PKA) pathways and the inhibition of $Ca^{2+}$-influx through L-type calcium channels. | Mahobiya et al., 2018 |

*EXCLI Journal 2020;19:627-634 – ISSN 1611-2156*
*Received: March 15, 2020, accepted: May 05, 2020, published: May 13, 2020*

| **Key findings** | **Reference** |
|---|---|
| The protective mechanism of kaempferol in mouse primary hepatocyte injury induced by ethanol has been reported to be due to its involvement in the synchronous, early and persistent inhibition of mitochondrial and microsomal cytochrome P450 2E1 (CYP2E1), cytosolic heat shock protein 70 (Hsp70), and nuclear and cytosolic specificity protein 1 (SP1). | Zhou et al., 2018 |
| Kaempferol may be considered a naturally occurring anti-diabetic compound that works by preventing glucose production and enhancing insulin sensitivity. The suppression of hepatic gluconeogenesis by kaempferol is considered to occur through its direct inhibitory action on the enzymatic activity of pyruvate carboxylase (PC) | Alkhalidy et al., 2018b |
| Kaempferol has been reported to delay the loss of climbing ability and memory, prevent oxidative stress, and inhibit acetylcholinesterase activity. Therefore, kaempferol may be utilized as a possible therapeutic agent to prevent the progression of AD. | Beg et al., 2018 |
| Both checkpoint kinase 2 (Chk2) and death receptors play important roles in the anticancer activity of kaempferol observed in A2780/CP70 cells. These findings provide more evidence for the anti-ovarian cancer properties of kaempferol and demonstrate that kaempferol could be used as a potential adjuvant therapy for ovarian cancer. | Gao et al., 2018 |
| Kaempferol can be used to act against γ-radiation-induced tissue damage and mortality via inhibiting oxidative stress and modulating apoptotic molecules both *in vivo* and *in vitro*. | Wang et al., 2018 |
| When using kaempferol for bone regeneration purposes it has been revealed that it reduces glucocorticoid-induced bone loss and enhanced bone regeneration at the fractured site, demonstrating the positive role of flavonoids on bone health. | Adhikary et al., 2018 |
| Kaempferol exhibits gastroprotective ability, which may occur by preserving gastric mucous glycoproteins levels, preventing neutrophil accumulation and myeloperoxidase activity, regulating the levels of pro-inflammatory cytokines, and enhancing nitric oxide (NO) production. | Li et al., 2018 |
| Kaempferol decreases sepsis-induced acute lung injury in mice by preventing the oxidative stress, inducible NO synthase (iNOS), and intercellular adhesion molecule 1 (ICAM-1) pathways. | Rabha et al., 2018 |
| Kaempferol reduces the lipid profile, infracted area, and oxidative stress in isoprenaline-induced myocardial injury in rats. | Vishwakarma et al., 2018 |
| Kaempferol prevents the migration and invasion of fibroblast-like synoviocytes (FLSs) in rheumatoid arthritis (RA) by suppressing the activation of the MAPK pathway without hampering the expression of TNF-α receptors. | Pan et al., 2018 |
| Kaempferol enhances apoptosis and impedes multidrug resistance in a concentration-dependent manner without any differential effect on leukemic cells. Therefore, kaempferol may be used as an appropriate alternative for all-trans retinoic acid (ATRA) in acute promyelocytic leukemia (APL) patients. | Moradzadeh et al., 2018 |
| Kaempferol can inhibit angiogenesis by suppressing hypoxia-inducible factor-1α (HIF-1α) and vascular endothelial growth factor receptor 2 (VEGFR2) activation via ERK/p38 MAPK and PI3K/Akt/mTOR signaling in endothelial cells. | Kim, 2017 |
| Kaempferol targets estrogen-related receptor α (ERRα) and prevents angiogenesis in human retinal endothelial cells (HRECs) under high glucose conditions. Therefore, kaempferol can be considered a potential drug for controlling the progression of diabetic retinopathy. | Wu et al., 2017 |
| Kaempferol imparts a starting point for the development of novel anti-biofilm drugs, which may decrease the risk of bacterial drug resistance, inhibiting *Staphylococcus aureus* biofilm-related infections. | Ming et al., 2017 |
| Apigenin, hesperidin, and kaempferol exhibit an anti-adipogenic and delipidating effect in human adipocytes derived from human mesenchymal stem cells (MSCs). | Gómez-Zorita et al., 2017 |

*Received: March 15, 2020, accepted: May 05, 2020, published: May 13, 2020*

| **Key findings** | **Reference** |
|---|---|
| Kaempferol helps to induce hepatocarcinoma cell death via an ER stress and CHOP-autophagy signaling pathway. Therefore, kaempferol may be used as a potential chemopreventive agent for patients with hepatocellular carcinoma. | Guo et al., 2017 |
| Kaempferol can prevent and reverse ventricular fibrosis and cardiac dysfunction affording an experimental basis for the clinical treatment on ventricular fibrosis. | Liu et al., 2017 |
| Kaempferol sensitizes ovarian cancer cells to tumor necrosis factor-related apoptosis-inducing ligand (TRAIL)-induced apoptosis by up-regulation of death receptors 4 and 5 (DR4 and DR5, respectively) through an ERK/JNK/CHOP pathway. | Zhao et al., 2017 |
| Kaempferol significantly reduces interleukin-1β-stimulated pro-inflammatory mediators in rat OA chondrocytes by preventing the activation of the NF-κB pathway. Therefore, kaempferol can be used as an anti-inflammatory and anti-arthritis agent. | Zhuang et al., 2017 |
| Kaempferol as a precursor increases coenzyme Q (Q) levels via combining its ability to upregulate sirtuin-3. Therefore, kaempferol is a potential candidate for the design of drugs aimed toward increasing endogenous Q biosynthesis, particularly in the kidneys. | Fernández-Del-Río et al., 2017 |
| Kaempferol can be applied to treat myocardial ischemia-reperfusion injury in diabetic rats by reducing AGE-RAGE/MAPK induced oxidative stress and inflammation. | Suchal et al., 2017 |
| Kaempferol reveals protective effects toward H9N2 virus-induced inflammation via the suppression of themyeloid differentiation factor 88 (TLR4/MyD88)-mediated NF-κB and MAPKs pathways. Therefore, kaempferol can be considered as an effectual drug used for the treatment of influenza virus-induced ALI. | Zhang et al., 2017 |
| Kaempferol exhibits a cytoprotective effect against the lipotoxic activation of autophagy via an AMPK/mTOR pathway. | Varshney et al., 2017 |
| Kaempferol can be utilized for the clinical treatment of uterine fibroids due to its inhibitory effect on the proliferation of uterine fibroids cells. | Li et al., 2017 |

## *Conflict of interest*

The authors declare no conflict of interest.

## Acknowledgements

This research was supported by Golden Seed Project (213006051WTE11) funded by the Ministry of Agriculture, Food and Rural Affairs (MAFRA), the Ministry of Oceans and Fisheries (MOF), the Rural Development Administration (RDA), and the Korea Forest Service (KFS), Republic of Korea.

## Conflict of interest

The authors declare no conflict of interest.

## Figures and Tables

**Table 1:**
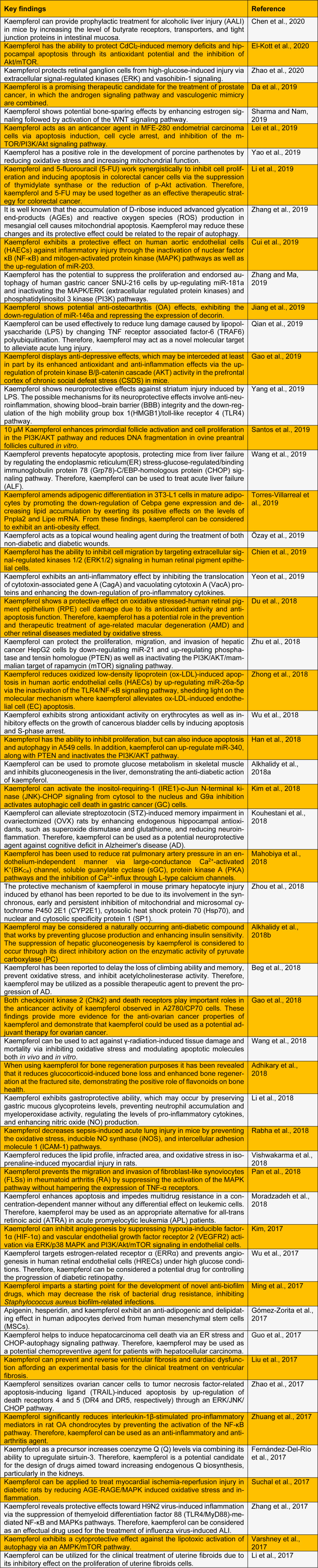
Recent studies on the biological and pharmacological activities of kaempferol
